# Taxonomic notes and key to the West Palearctic *Antocha* (*Antocha*) Osten Sacken, 1860 (Diptera, Limoniidae) with description of a new species from Morocco

**DOI:** 10.3897/BDJ.11.e103849

**Published:** 2023-07-03

**Authors:** Youness Mabrouki, Andrei Bogdan Terec, Fouzi A. Taybi, Anna Dénes, Lujza Keresztes

**Affiliations:** 1 Sidi Mohamed Ben Abdellah University, Faculty of Sciences Dhar El Mehraz, Biotechnology, Conservation and Valorisation of Natural Resources Laboratory, Fez. B.P. 1796, 30003, Fez, Morocco Sidi Mohamed Ben Abdellah University, Faculty of Sciences Dhar El Mehraz, Biotechnology, Conservation and Valorisation of Natural Resources Laboratory, Fez. B.P. 1796, 30003 Fez Morocco; 2 Doctoral School of Integrative Biology, Babeş-Bolyai University, Republicii 44, 400015, Cluj-Napoca, Romania Doctoral School of Integrative Biology, Babeş-Bolyai University, Republicii 44, 400015 Cluj-Napoca Romania; 3 Centre 3B, Laboratory of Advance Hydrobiology and Biomonitoring, Babeș-Bolyai University, Clinicilor 5-7, 400006, Cluj-Napoca, Romania Centre 3B, Laboratory of Advance Hydrobiology and Biomonitoring, Babeș-Bolyai University, Clinicilor 5-7, 400006 Cluj-Napoca Romania; 4 Université Mohammed Premier, Faculté Pluridisciplinaire de Nador, Équipe de Recherche en Biologie et Biotechnologie Appliquées, B.P 300, 62700 Selouane, Morocco, Oujda, Morocco Université Mohammed Premier, Faculté Pluridisciplinaire de Nador, Équipe de Recherche en Biologie et Biotechnologie Appliquées, B.P 300, 62700 Selouane, Morocco Oujda Morocco

**Keywords:** Antocha (Antocha), conservation, freshwater ecosystems, Mediterranean area, new species, the Middle Atlas region

## Abstract

**Background:**

The Mediterranean Region of the West Palearctic is one of the most species-rich biomes in the world, hosting a high level of endemism and relict species with important conservation value. The North Africa Atlas Mountains (spanning Morocco, Algeria and Tunisia) belong to a poorly-investigated region of the Mediterranean area, with overlooked aquatic biodiversity; hence, a number of species still remain to be discovered.

**New information:**

The subgenus Antocha (Antocha) Osten Sacken, 1860 is recorded for the first time from Africa, with a description of A. (A.) staryi Keresztes & Mabrouki **sp. nov.** from hilly regions of the Middle Atlas region, Morocco. The unique design of the male terminalia differentiates well the newly-discovered species from its closely-related and range-restricted A. (A.) phoenicia Thomas and Dia, 1982. This is in contrast with the high intraspecific and geographically poorly-defined variability of the widespread A. (A.) vitripennis (Meigen, 1830), for which morphological variability of male genital structures is discussed.

Illustrations of male genital parts, distribution data and key to the species from the West Palearctic area are also provided.

## Introduction

The Mediterranean area is one of the world’s outstanding biodiversity hotspots with high conservation value within the West Palearctic (Delibes-Mateos et al. 2008). The geographic area surrounding the Mediterranean Sea hosts a unique biodiversity resulting from long-term evolutionary events in both terrestrial and aquatic ecosystems, with high numbers of relicts and/or endemic taxa, facing, nowadays, severe habitat loss and decline of some species (Barredo et al. 2016). The southern region of the Mediterranean area, including Morocco, has a special interest in nature conservation, because its geographical position, localised along the coastlines of northern Africa between the Atlantic Ocean and the Mediterranean Sea, supports high diversity ecosystems shaped by both the Mediterranean and the Saharan climate types (Abdul Malak et al. 2010). Despite its geographic position and extraordinary diversity of ecosystems, Morocco has been poorly investigated in general, especially regarding its aquatic Diptera fauna, including short-palped craneflies, Limoniidae (Pierre 1924, Séguy 1941a, Séguy 1941b, Vaillant 1956, Starý 1971, Starý 1974, Savchenko et al. 1992, Starý 2006, Pârvu et al. 2006, Starý and Oosterbroek 2008, Starý 2009, Starý 2011, Driauach et al. 2013, Driauach and Belqat 2015, Driauach and Belqat 2016, Adghir et al. 2018, Ebejer et al. 2019, Eiroa et al. 2020, de Jong et al. 2021, Taybi et al. 2021, Kettani et al. 2022).

*Antocha* Osten Sacken, 1860 is a relatively large genus of the subfamily Limoniinae (Diptera, Limoniidae), including 161 species according to Oosterbroek (2023), grouped into three subgenera, *Antocha* Osten Sacken, 1860, *Orimargula* Mik, 1883 and *Proantocha* Alexander, 1919. Species of this genus are distributed in almost all zoogeographical regions of the world, with high species diversity detected in the Oriental (83 species) and the East Palearctic (53 species) areas. A lower number of species was recorded from the Afrotropics (21 species), Nearctic (7 species), Australasian/Oceanian (3 species) and Neotropics (1 species). In the West Palaearctic Region, members of only two subgenera occur. The subgenus Antocha is represented by four species, frequently collected in lower mountainous to hilly areas, while A. (Orimargula) has a single species, distributed mostly in the mountains belonging to this biogeographic area.

The subgenus Antocha contains small to medium-sized dipterans with body length ranging between 2.8 and 5.0 mm, up to 8.0 mm (Podenas and Byun 2013) and exceptionally up to 11 mm in the case of A. (A.) unicollis Alexander, 1968. A general revision of the species belonging to the subgenus was recently published by Podenas and Byun (2013), Podenas and Byun (2014), Podenas and Young (2015), Markevičiūté et al. (2019), Markevičiūté et al. (2021) who focused more on the Oriental fauna. The general colour of the species is brownish, brownish-yellow to dark brown or nearly black. The most important morphological characters of the species of A. (Antocha) are the following: antennae simple with rings of verticils at bases of flagellomeres or antennal flagellomeres short-oval and covered with long dense pubescence (Podenas and Byun 2013); wings are wide with large, well developed, prominent anal angle; male terminalia slightly wider than abdomen, gonocoxite elongate, oval or cylindrical and often with well-developed ventro-mesal lobe; two pairs of terminal gonostyli, with outer gonostylus usually darkened and strongly sclerotised and the inner gonostylus usually fleshy and curved downwards; ovipositor with a straight or slightly arched cercus, with the lower margin smooth or serrated (Podenas and Byun 2014, Podenas and Young 2015, Markevičiūté et al. 2019). The four West Palaerctic species included in this subgenus are: A. (A.) hirtipes Savchenko, 1971 (recorded from Georgia, only); A. (A.) libanotica Lackschewitz, 1940 (recorded from Georgia, Armenia, Azerbaijan, Cyprus, Lebanon, Iran, Turkmenistan, Tajikistan and Afghanistan), A. (A.) phoenicia Thomas and Dia, 1982 (only recorded from Lebanon) and A. (A.) vitripennis Meigen, 1830, widespread in the whole West Palearctic area and also recorded from the East Palearctic Region (Oosterbroek 2023).

Larvae are aquatic, apneustic, developing in the riffles, gravel or detritus of fast running streams and rivers (Fuller and Hynes 1987, Ujvárosi 2005, Vogtenhuber 2007). Adults are present in large numbers close to the larvae sites near the water margins and they are usually attracted to light or fall into Malaise-traps in suitable locations (Keresztes, pers. data). Hancock and Hewitt (2020) reported also a curious case of a large number of adult specimens trapped by the glandulous leaf of the butterwort plant *Pinguicularia* ssp. near the river margins in Spain.

## Materials and methods

**Figure 1.**
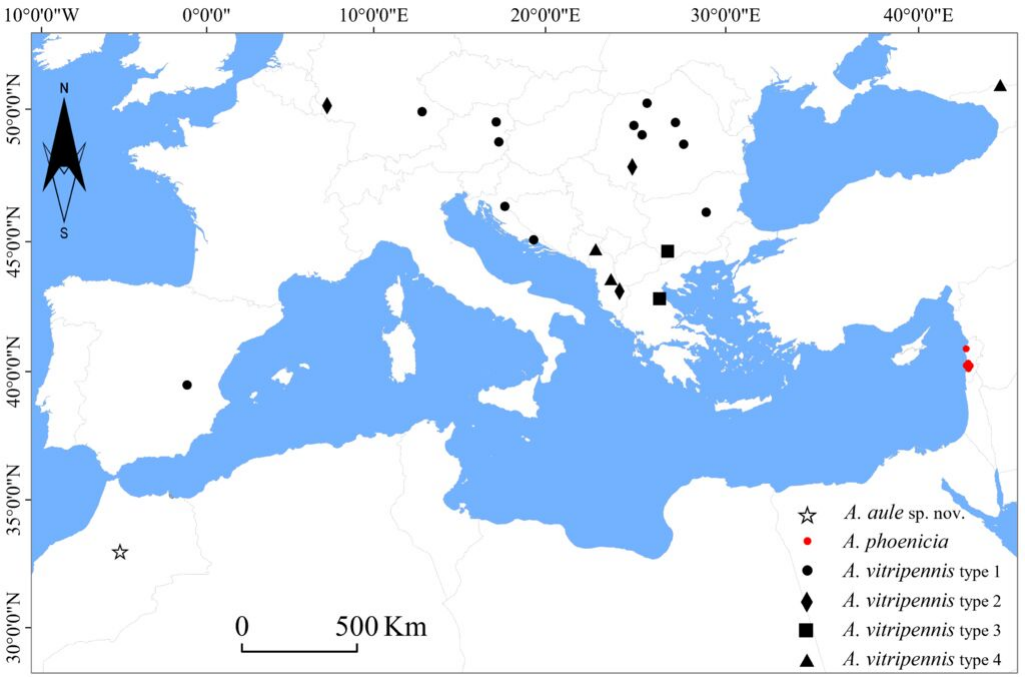
Distribution data of Antocha (Antocha) species included in the present investigation.

Material included in this study represent 201 male individuals of the subgenus A. (A.), including the new species. Adult specimens were collected between 2001 and 2022 using entomological nets and light traps, with a large amount of material collected by entomologists from different parts of the West Palearctic Region (Fig. 1). All the material listed here is stored in 96% ethanol and deposited in the Diptera Collection of the Faculty of Biology and Geology, Babeș-Bolyai University, Cluj-Napoca, Romania. Holotype and paratypes of the new species are deposited in the Museum of Zoology of the Babeș-Bolyai University (MZBBU), Cluj-Napoca, Romania. Male genitalia were left overnight in 10% potas­sium hydroxide (KOH) and for one hour in undiluted glacial acetic acid, to neutralise and wash out the soap that was created from the soft tissues. The male genitalia were then transferred to a larger amount of glycerol to wash out the acid. Afterwards, they were transferred to a drop of glycerol on a slide with rounded excavation. Male abdomens were dissected and examined under a microscope (Olympus CZ23). Photos were taken using a Canon EOS 650D digital camera, attached to the microscope, with an LM Digital SLR Adapter (MicroTechLab, Austria). Layer photos were combined using Zerene Stacker software. Nomenclature of the genital parts follows Kato and Tachi (2019) and Lv et al. (2023).

## Taxon treatments

### Antocha (Antocha) staryi

Keresztes & Mabrouki sp. nov.

EA759596-275E-5948-A68A-BEFD79916E53

8F608A95-F4AA-443C-8634-4FA00F05FAAF

#### Materials

- **Type status:** Holotype. **Occurrence:** sex: 1 male; lifeStage: adult; occurrenceID: CB927E68-C824-5E85-8FAE-964FC5DE2F27; **Taxon:** scientificNameID: urn:lsid:zoobank.org:pub:8F608A95-F4AA-443C-8634-4FA00F05FAAF; class: Insecta; order: Diptera; family: Limoniidae; genus: Antocha; subgenus: Antocha; **Location:** continent: Africa; waterBody: Cascade near Bakrit; country: Morocco; locality: Middle Atlas Range, Bakrit; verbatimElevation: 1640; verbatimSRS: WGS84; decimalLatitude: 33.049438; decimalLongitude: -5.272774; **Identification:** identifiedBy: Keresztes, L.; **Event:** eventDate: 16-05-2021; eventRemarks: leg. Mabrouki, Y. & Taybi F.A.
- **Type status:** Paratype. **Occurrence:** sex: 2 males, 4 females; lifeStage: adult; occurrenceID: D03EBE02-2C09-573C-8C87-2E409459F84C; **Taxon:** scientificNameID: urn:lsid:zoobank.org:pub:8F608A95-F4AA-443C-8634-4FA00F05FAAF; class: Insecta; order: Diptera; family: Limoniidae; genus: Antocha; subgenus: Antocha; **Location:** continent: Africa; waterBody: Cascade near Bakrit; country: Morocco; locality: Middle Atlas Range, Bakrit; verbatimElevation: 1640; verbatimSRS: WGS84; decimalLatitude: 33.049438; decimalLongitude: -5.272774; **Identification:** identifiedBy: Keresztes, L.; **Event:** eventDate: 16-05-2021; eventRemarks: leg. Mabrouki, Y. & Taybi F.A.

#### Description

**Figure 2.**
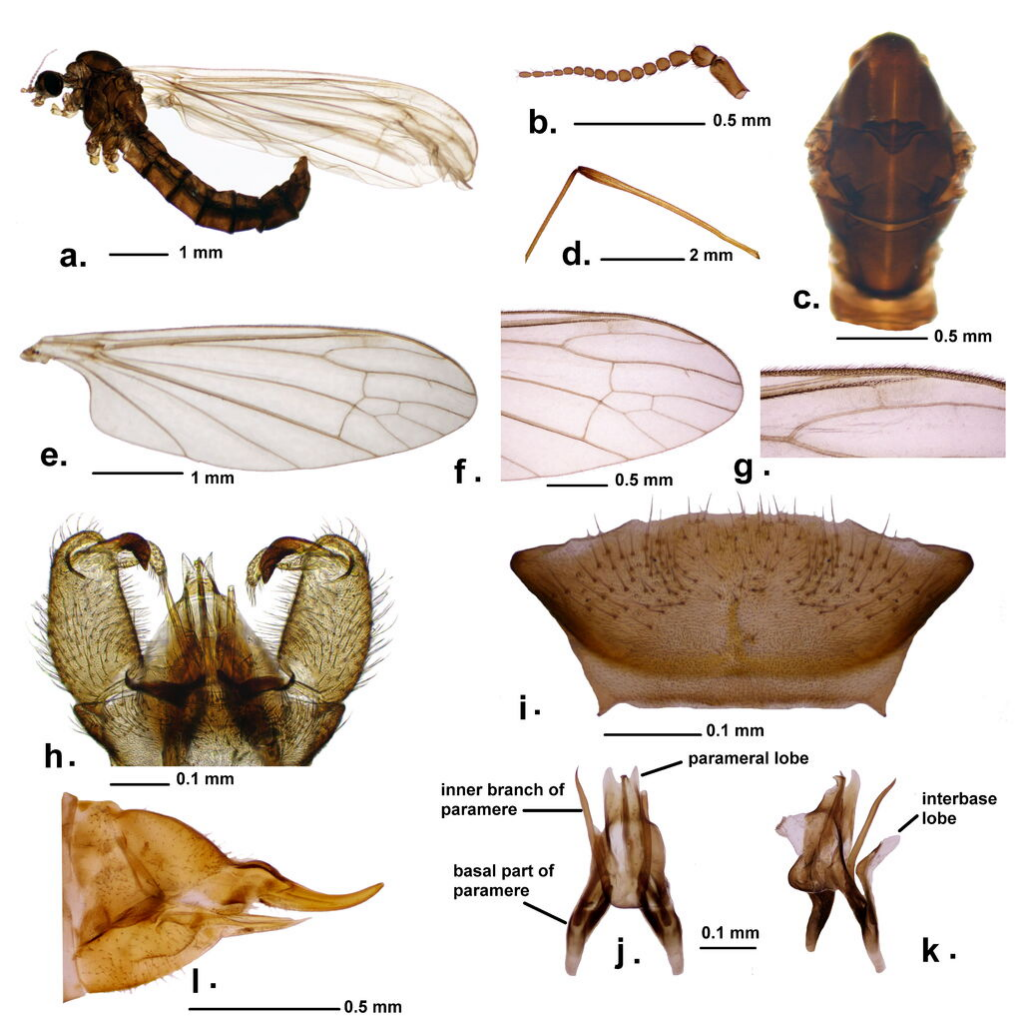
Antocha (Antocha) staryi sp. nov. **a** male habitus, lateral view; **b** antenna; **c** thorax, dorsal view; **d** femur and part of tibiae, third left leg; **e** right wing; **f** tip of the right wing; **g** stigma on the right wing; **h** male hypopygium, dorsal view; **i** tergite 9, dorsal view; **j** aedeagal complex, dorsal view; **k** aedeagal complex, lateral view; **l** female terminalia, lateral view.

**Male**. Colour dark brown. Body length 5.3‒5.5 mm, wing length 6.2‒6.5 mm. General appearance as in Fig. 2a.

*Head*. Dark brown, with light brown rostrum. Palpus greyish-brown. Antennae brownish, with 16 segments (Fig. 2b). Scape long and cylindrical. Pedicel is large and short. Antennal flagellomeres short-oval and covered with long dense pubescence, except the last four segments which gradually become smaller and cylindrical, the last segment ending pointed. Short bristles present on every antennal segment, rings of verticils at bases of flagellomeres, mostly on its dorsal surface. Larger bristles are also present on the ventral surface of segments 13, 15 and 16.

*Thorax*. Prescutum with anterolateral region yellowish. Prescutum and presutural scutum with a broad brown median and two broad lateral bands (Fig. 2c). The lateral bands extending to scutellum. Basal third of presutural scutum with conspicuous dark brown triangle. Scutellum greyish-brown. The rest of the thorax lighter brownish. Legs long and slender, pubescent (Fig. 2d). Coxae lighter to yellowish, rest of the legs brownish. Wing nearly translucent to whitish, generally white and large, with nearly right-angled cell a2 (Fig. 2e). Veins brownish-yellowish, with stronger bristles on C and R1 (Fig. 2e, f and g). Stigma indistinct, nearly invisible (Fig. 2f and g). Halter with a yellowish stem and a whitish-greyish knob.

*Abdomen*. Dark brown, first segment lighter, with whitish-clouded tint dorsally, the last segments darker.

*Male terminalia*. Tergite 9 in the shape of transverse plate with posterior margin straight (Fig. 2i). Posterolateral angle extended, nearly triangular with darkened margin. Gonocoxite cylindrical, with long setae (Fig. 2h). Outer gonostylus strongly sclerotised, short and curved. Distal end shorter and thicker and less pointed with a hardly visible hump on the dorsal surface. Inner gonostylus fleshy, pubescent, distal end abruptly curved downwards. Aedeagal complex with interbase exteriorly-diverged (Fig. 2k). Inner branch of parameres straight and parallel, slightly bent near the apex (Fig. 2j). Parameres with basal parts stout and divergent, apical parts forming two parameral lobes on either side of aedeagus. Lateral edges of the lobes straight, tips pointed directed straight back (Fig. 2h, j and k).

**Female.** Colour similar to males. Body length 5.8 mm, wing length 6.7 mm. General habitus similar to males, except the dark brown triangle on metathorax reduced.

Ovipositor. Cerci and hypogynal valve long and slender (Fig. 2l).

#### Diagnosis

Antocha (A.) staryi sp. nov. can be recognised by dark brown body colouration, thoracic dark brown triangle, strongly sclerotised and curved outer gonostylus and tergite 9 having straight posterior margin and darkened, nearly triangular posterolateral lobes. Aedeagal complex with inner branch of parameres long, parameres apically forming slightly curved lobes.

The closest regional ally is A. (A.) phoenicia. Both species are similar in general features with A. (A.) vitripennis and are characterised by the long inner branch of the paramere, similar shape of parameral lobe and tergite 9 possessing extended posterolateral lobe. Still A. (A.) staryi sp. nov. can be separated from A. (A.) phoenicia by the unique apical part of the paramere (the parameral lobe) which is sharply narrowed to the distal end, longer and curved ventrally and also by details of tergite 9, with the posterior margin having a prominent and angular middle region in A. (A.) phoenicia, but less prominent, more or less straight in A. (A.) staryi sp. nov., but with posterolateral angle well developed, nearly triangular.

Antocha (A.) staryi sp. nov. is highly divergent from the European widespread A. (A.) vitripennis, as well as, in a series of morphological details in male genital structures, like the more or less straight posterior margin of tergite 9, but more sinuous in A. (A.) vitripennis, with a middle depression and two lateral humps. The exteriorly curved interbase lobe is well developed in A. (A.) staryi sp. nov., but less in A. (A.) vitripennis. Inner branch of the parameres parallel with aedeagus in A. (A.) staryi sp. nov., but highly divergent and sinuous in A. (A.) vitripennis. The apical part of the paramere with narrow and distally pointed parameral lobe in A. (A.) staryi sp. nov., but with a generally rounded lobe in A. (A.) vitripennis.

#### Etymology

Named after the outstanding cranefly taxonomist Jaroslav Starý, honouring his 80^th^ birth anniversary. A noun in genitive singular.

#### Distribution

The type locality of the new species, Bakrit, belongs to the Middle Atlas Range, which is located in the southwest to northeast of the Mediterranean part of Morocco. The scientific and socio-economic interests of the included aquatic ecosystems are no longer demonstrated as an area with rich and varied natural resources, which generally support the presence of an interesting aquatic biodiversity with high rates of endemism (Taybi et al. 2020, Ibrahimi et al. 2021, Mabrouki et al. 2021). The newly-discovered species from here complete the diversity of aquatic fauna from this location and support conservation efforts for these unique aquatic ecosystems.

#### Ecology

**Figure 3.**
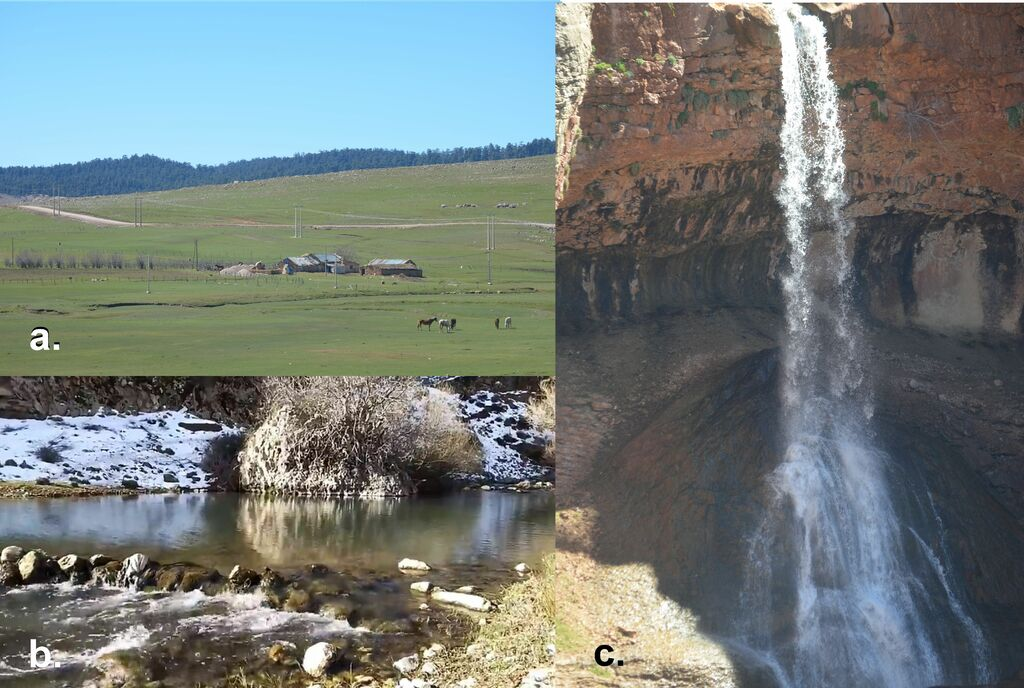
Different landscape elements of the Bakrit region, Middle Atlas, Morocco, showing the habitat of the Antocha (Antocha) staryi sp. nov. **a** general view of the area; **b** detail on the Oum Errabiâ River; **c** waterfall on the Oum Errabiâ River.

The species was discovered in a single site in Morocco; therefore, we consider a range-restricted micro-endemic species of the Middle Atlas Range. Specimens were captured in Bakrit, a location in the Middle Atlas Range, a mountain range stretching over some 350 km, from southwest to northeast of the Mediterranean part of Morocco, located between the Rif Mountains and the High Atlas Mountains and covering a total area of 2.3 million hectares, i.e. 18% of Morocco’s high altitude mountain domain (Fig. 3a). This chain belongs to the Atlas Mountains and more precisely, to one of the three elements of the Moroccan Atlas, the other two being the High Atlas and the Anti-Atlas (or Lesser Atlas). The heavy rainfall gives the Middle Atlas Mountains the form of a “water tower” (also via snowmelt hydrology) from both hydrogeological and hydrographic perspectives and is the main water supply for median and low reaches.

Bakrit Region is well-known for its rich superficial water resources, i.e. streams, waterfalls and springs and the new species was captured from the banks of a fast-flowing stream, with the type locality belonging to the Oum Errabiâ River Basin (Fig. 3b and c).

##### Accompanying species

Different aquatic invertebrate species can be found in the habitat of A. (A.) staryi sp. nov. including the recently-described caddisfly *Tinodesatlasensis* Ibrahimi, Mabrouki & Taybi, 2021 and freshwater gastropod *Pseudamnicolabouhaddiouii* Taybi, Glöer & Mabrouki, 2022, in addition to other invertebrate species, such as *Hydropsyche* sp. (Trichoptera); *Ecdyonurusrothschildi* (Navàs, 1929), *Rhithrogena* sp., *Baetis* sp., *Caenis* sp. (Ephemeroptera); *Calopteryxhaemorrhoidalis* (Vander Linden, 1825), *Anax* sp., *Onychogomphus* sp., *Sympetrum* sp., *Orthetrum* sp. (Odonata); *Hydrometrastagnorum* (Linnaeus, 1758) (Heteroptera); *Atyaephyradesmarestii* (Millet, 1831) (Decapoda); *Physellaacuta* (Draparnaud, 1805), *Ancylusfluviatilis* O.F. Müller, 1774; *Theodoxus* sp., *Melanopsis* sp. (Mollusca); *Simulium* sp., *Prosimulium* sp. (Diptera) (Ibrahimi et al. 2021, Taybi et al. 2022).

#### Antocha (Antocha) vitripennis (Meigen, 1830)

*Limnobiavitripennis* (Meigen, 1830) (type locality not given, ? near Stolberg, Germany Meigen (1830), Savchenko et al. (1992). Syn. *Antochaopalizans* auct. nec *opalizans* Osten Sacken, 1859: 220 (Geiger 1985).

Synonyms: *Antochaobscura* Strobl, 1906 (as variety of *A.opalizans*, type-locality Ronda, Spain), *Antochafulvescens* Lackschewitz, 1940 (type locality Vernet-les-Bains, France, Savchenko et al. (1992)), synonymised with *A.vitripennis* by Geiger (1985).

**Morphological variability of male terminalia of A. (A.) vitripennis (Meigen, 1830) in the West Palearctic Region**

**Figure 4.**
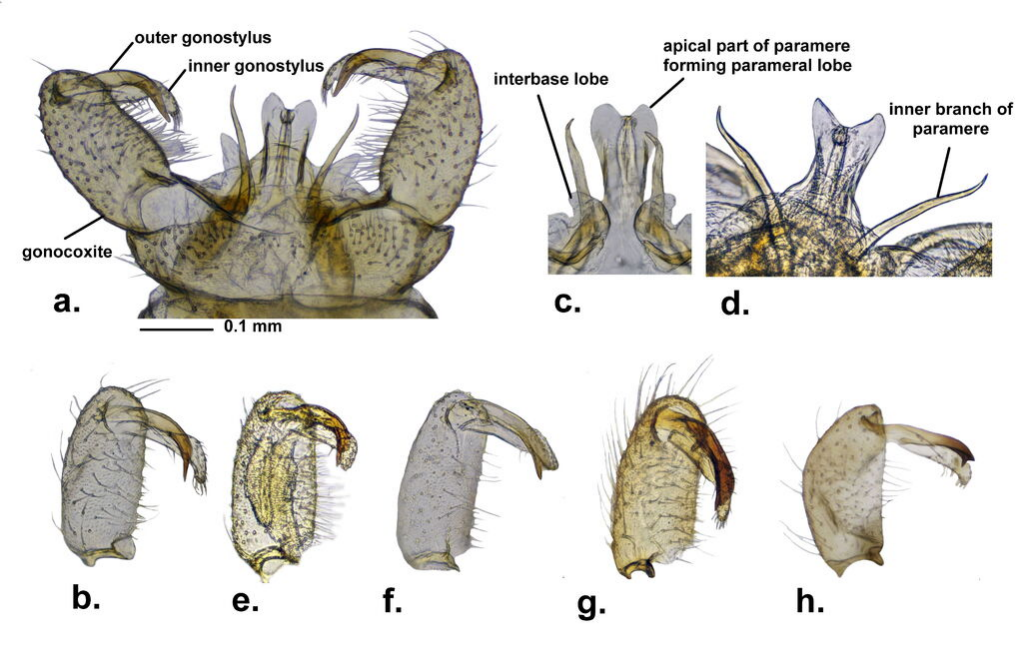
Variability of the distal end of the outer gonostylus of the male genital structures of Antocha (Antocha) vitripennis from the West Palearctic area: **a** male terminalia, dorsal (Regensburg, Germany); **b** gonocoxite with outer gonostylus of type 1 (Germany); **c** type 1, aedeagus with parallel inner branch of parameres (Germany); **d** type 1, aedeagus with divergent inner branch of parameres (Austria); **e** gonocoxite with outer gonostylus of type 2 (Germany, Kassel); **f** gonocoxite with outer gonostylus of type 3 (Greece, Olympus); **g** gonocoxite with outer gonostylus of type 4 (Montenegro, Prokletje Mountains); **h** gonocoxite with outer gonostylus, special case of type 2 (Albania, Korce).

A number of 198 male individuals of *A.vitripennis* were investigated from the whole West Palearctic Region, representing 20 different populations from Albania, Austria, Bulgaria, Croatia, Georgia, Germany, Greece, Montenegro, Romania and Spain (Table 1). A comparative analysis of the outer gonostylus, inner branch of parameres and parameral lobes of individual specimens shows high variability in the species within its whole distribution area, which allowed us to group individuals in at least four different morphological types (Fig. 4).

### Antocha (Antochа) vitripennis

(Meigen, 1830)

A5F73C22-DB94-52AE-A981-1E04380A3A4C

#### Materials

- **Type status:** Other material. **Occurrence:** sex: 25 males; lifeStage: adult; occurrenceID: 79366A14-4113-5B33-A33D-F8A53400A6F5; **Taxon:** class: Insecta; order: Diptera; family: Limoniidae; genus: Antocha; taxonRemarks: Antocha (A.) vitripennis, type 1; **Location:** continent: Europe; country: Austria; locality: Burgenland; verbatimSRS: WGS84; decimalLatitude: 47.373709; decimalLongitude: 16.010401; **Identification:** identifiedBy: Keresztes, L.; **Event:** eventDate: 19-06-2012; eventRemarks: leg. Graf, W.
- **Type status:** Other material. **Occurrence:** sex: 5 males; lifeStage: adult; occurrenceID: 9DDFE354-5E4F-573E-942B-3843C0C0DB9B; **Taxon:** class: Insecta; order: Diptera; family: Limoniidae; genus: Antocha; taxonRemarks: Antocha (A.) vitripennis, type 1; **Location:** continent: Europe; country: Austria; locality: Wienna hills; verbatimSRS: WGS84; decimalLatitude: 48.136847; decimalLongitude: 16.098223; **Identification:** identifiedBy: Keresztes, L.; **Event:** eventDate: 15-06-2012; eventRemarks: leg. Graf, W.
- **Type status:** Other material. **Occurrence:** sex: 4 males; lifeStage: adult; occurrenceID: 6A29D2D0-E4AF-5CE3-A81C-036A4BD20558; **Taxon:** class: Insecta; order: Diptera; family: Limoniidae; genus: Antocha; taxonRemarks: Antocha (A.) vitripennis, type 1; **Location:** continent: Europe; country: Bulgaria; locality: Stara Planina; verbatimSRS: WGS84; decimalLatitude: 42.784125; decimalLongitude: 25.912326; **Identification:** identifiedBy: Keresztes, L.; **Event:** eventDate: 01-06-2013; eventRemarks: leg. Kolcsár, L.-P. & Török E.
- **Type status:** Other material. **Occurrence:** sex: 7 males; lifeStage: adult; occurrenceID: 2A66D0CC-CCD5-5A6E-9A20-A6D9334F252B; **Taxon:** class: Insecta; order: Diptera; family: Limoniidae; genus: Antocha; taxonRemarks: Antocha (A.) vitripennis, type 1; **Location:** continent: Europe; country: Croatia; locality: NP Plitvicka Jezera; verbatimSRS: WGS84; decimalLatitude: 44.925833; decimalLongitude: 15.619167; **Identification:** identifiedBy: Keresztes, L.; **Event:** eventDate: 30-06-2008; eventRemarks: leg. Ivkovic, M.
- **Type status:** Other material. **Occurrence:** sex: 6 males; lifeStage: adult; occurrenceID: 251B5C15-3723-56FD-B042-0C01B7967645; **Taxon:** class: Insecta; order: Diptera; family: Limoniidae; genus: Antocha; taxonRemarks: Antocha (A.) vitripennis, type 1; **Location:** continent: Europe; country: Croatia; locality: Omiši Forests; verbatimSRS: WGS84; decimalLatitude: 43.437297; decimalLongitude: 16.757657; **Identification:** identifiedBy: Keresztes, L.; **Event:** eventDate: 19-07-2005; eventRemarks: leg. Ivkovic, M.
- **Type status:** Other material. **Occurrence:** sex: 2 males; lifeStage: adult; occurrenceID: BECE70D5-B754-5935-9001-B81960BA27E7; **Taxon:** class: Insecta; order: Diptera; family: Limoniidae; genus: Antocha; taxonRemarks: Antocha (A.) vitripennis, type 1; **Location:** continent: Europe; country: Germany; locality: Danube lowland; verbatimSRS: WGS84; decimalLatitude: 49.026846; decimalLongitude: 12.075860; **Identification:** identifiedBy: Keresztes, L.; **Event:** eventDate: 13-07-2013; eventRemarks: leg. Graf, W.
- **Type status:** Other material. **Occurrence:** sex: 22 males; lifeStage: adult; occurrenceID: D66D3EC9-E42A-5987-97F1-822CA33263A1; **Taxon:** class: Insecta; order: Diptera; family: Limoniidae; genus: Antocha; taxonRemarks: Antocha (A.) vitripennis, type 1; **Location:** continent: Europe; country: Romania; locality: Rodnei Mts.; verbatimSRS: WGS84; decimalLatitude: 47.423995; decimalLongitude: 24.548807; **Identification:** identifiedBy: Keresztes, L.; **Event:** eventDate: 26-07-2003; eventRemarks: leg. Keresztes, L.
- **Type status:** Other material. **Occurrence:** sex: 33 males; lifeStage: adult; occurrenceID: DEDA028B-7B5B-55EF-8CA3-E2921DA7F57E; **Taxon:** class: Insecta; order: Diptera; family: Limoniidae; genus: Antocha; taxonRemarks: Antocha (A.) vitripennis, type 1; **Location:** continent: Europe; country: Romania; locality: Harghita Mts.; verbatimSRS: WGS84; decimalLatitude: 46.412488; decimalLongitude: 25.745437; **Identification:** identifiedBy: Keresztes, L.; **Event:** eventDate: 15-07-2001; eventRemarks: leg. Keresztes, L.
- **Type status:** Other material. **Occurrence:** sex: 37 males; lifeStage: adult; occurrenceID: 2B539852-8D49-5E50-92FF-60D19C01A2FA; **Taxon:** class: Insecta; order: Diptera; family: Limoniidae; genus: Antocha; taxonRemarks: Antocha (A.) vitripennis, type 1; **Location:** continent: Europe; country: Romania; locality: Ciucaș Mts.; verbatimSRS: WGS84; decimalLatitude: 45.534297; decimalLongitude: 25.836821; **Identification:** identifiedBy: Keresztes, L.; **Event:** eventDate: 21-07-2004; eventRemarks: leg. Keresztes, L.
- **Type status:** Other material. **Occurrence:** sex: 7 males; lifeStage: adult; occurrenceID: AC7E5A4E-0B19-5CB6-8FE1-CC1ADB71D099; **Taxon:** class: Insecta; order: Diptera; family: Limoniidae; genus: Antocha; taxonRemarks: Antocha (A.) vitripennis, type 1; **Location:** continent: Europe; country: Romania; locality: Trascaului Mts.; verbatimSRS: WGS84; decimalLatitude: 46.565262; decimalLongitude: 23.675828; **Identification:** identifiedBy: Keresztes, L.; **Event:** eventDate: 06-06-2001; eventRemarks: leg. Keresztes, L.
- **Type status:** Other material. **Occurrence:** sex: 8 males; lifeStage: adult; occurrenceID: 95346811-C0F2-5416-B99C-D741EE62D525; **Taxon:** class: Insecta; order: Diptera; family: Limoniidae; genus: Antocha; taxonRemarks: Antocha (A.) vitripennis, type 1; **Location:** continent: Europe; country: Romania; locality: Clujului Hills; verbatimSRS: WGS84; decimalLatitude: 46.755529; decimalLongitude: 23.509461; **Identification:** identifiedBy: Keresztes, L.; **Event:** eventDate: 24-08-2004; eventRemarks: leg. Keresztes, L.
- **Type status:** Other material. **Occurrence:** sex: 1 male; lifeStage: adult; occurrenceID: B87A4D3A-E5F6-5EBB-8754-ECF5CC2BAE6E; **Taxon:** class: Insecta; order: Diptera; family: Limoniidae; genus: Antocha; taxonRemarks: Antocha (A.) vitripennis, type 1; **Location:** continent: Europe; country: Spain; locality: Serrania de Cuenca; verbatimSRS: WGS84; decimalLatitude: 39.503257; decimalLongitude: -1.900691; **Identification:** identifiedBy: Keresztes, L.; **Event:** eventDate: 04-08-2009; eventRemarks: leg. Martinez, J.
- **Type status:** Other material. **Occurrence:** sex: 1 male; lifeStage: adult; occurrenceID: 0DBE5507-F359-5382-A5FE-55C18D749170; **Taxon:** class: Insecta; order: Diptera; family: Limoniidae; genus: Antocha; taxonRemarks: Antocha (A.) vitripennis, type 2; **Location:** continent: Europe; country: Albania; locality: Korce; verbatimSRS: WGS84; decimalLatitude: 41.016258; decimalLongitude: 20.513618; **Identification:** identifiedBy: Keresztes, L.; **Event:** eventDate: 03-05-2019; eventRemarks: leg. Keresztes, L.
- **Type status:** Other material. **Occurrence:** sex: 12 males; lifeStage: adult; occurrenceID: 460335F1-D823-5A08-B806-95B25AA9EBB1; **Taxon:** class: Insecta; order: Diptera; family: Limoniidae; genus: Antocha; taxonRemarks: Antocha (A.) vitripennis, type 2; **Location:** continent: Europe; country: Germany; locality: Eifel hills; verbatimSRS: WGS84; decimalLatitude: 49.755204; decimalLongitude: 6.735459; **Identification:** identifiedBy: Keresztes, L.; **Event:** eventDate: 05-08-2007; eventRemarks: leg. Neu, P.
- **Type status:** Other material. **Occurrence:** sex: 6 males; lifeStage: adult; occurrenceID: 7BB55E4A-2ABA-5593-9558-7DA78E32C417; **Taxon:** class: Insecta; order: Diptera; family: Limoniidae; genus: Antocha; taxonRemarks: Antocha (A.) vitripennis, type 2; **Location:** continent: Europe; country: Romania; locality: Cerna Mts; verbatimSRS: WGS84; decimalLatitude: 45.256819; decimalLongitude: 22.814289; **Identification:** identifiedBy: Keresztes, L.; **Event:** eventDate: 10-08-2004; eventRemarks: leg. Bálint, M.
- **Type status:** Other material. **Occurrence:** sex: 2 males; lifeStage: adult; occurrenceID: EBFCE991-C43B-5700-AED9-87D6EC9239F8; **Taxon:** class: Insecta; order: Diptera; family: Limoniidae; genus: Antocha; taxonRemarks: Antocha (A.) vitripennis, type 3; **Location:** continent: Europe; country: Bulgaria; locality: Pirin Mts; verbatimSRS: WGS84; decimalLatitude: 41.768952; decimalLongitude: 23.426897; **Identification:** identifiedBy: Keresztes, L.; **Event:** eventDate: 19-08-2003; eventRemarks: leg. Pauls, S.
- **Type status:** Other material. **Occurrence:** sex: 1 male; lifeStage: adult; occurrenceID: 923E950B-71DF-5477-8CEF-6679A1C8E175; **Taxon:** class: Insecta; order: Diptera; family: Limoniidae; genus: Antocha; taxonRemarks: Antocha (A.) vitripennis, type 3; **Location:** continent: Europe; country: Greece; locality: Olympus Mts.; verbatimSRS: WGS84; decimalLatitude: 40.079097; decimalLongitude: 22.373471; **Identification:** identifiedBy: Keresztes, L.; **Event:** eventDate: 14-07-2012; eventRemarks: leg. Rákosy, L.
- **Type status:** Other material. **Occurrence:** sex: 13 males; lifeStage: adult; occurrenceID: E1F6DA3B-BC17-5006-8729-BBFE1E25620C; **Taxon:** class: Insecta; order: Diptera; family: Limoniidae; genus: Antocha; taxonRemarks: Antocha (A.) vitripennis, type 4; **Location:** continent: Europe; country: Albania; locality: Domosdova Plain; verbatimSRS: WGS84; decimalLatitude: 41.075075; decimalLongitude: 20.498387; **Identification:** identifiedBy: Keresztes, L.; **Event:** eventDate: 01-05-2019; eventRemarks: leg. Keresztes, L.
- **Type status:** Other material. **Occurrence:** sex: 1 male; lifeStage: adult; occurrenceID: 15613FC2-197C-5AEA-AD73-E26DB524C721; **Taxon:** class: Insecta; order: Diptera; family: Limoniidae; genus: Antocha; taxonRemarks: Antocha (A.) vitripennis, type 4; **Location:** continent: Europe; country: Georgia; locality: North Caucaus; verbatimSRS: WGS84; decimalLatitude: 43.122052; decimalLongitude: 42.750700; **Identification:** identifiedBy: Keresztes, L.; **Event:** eventDate: 18-07-2012; eventRemarks: leg. Graf, W.
- **Type status:** Other material. **Occurrence:** sex: 5 males; lifeStage: adult; occurrenceID: 7E92A44E-7B20-570D-97A9-C782E16951D0; **Taxon:** class: Insecta; order: Diptera; family: Limoniidae; genus: Antocha; taxonRemarks: Antocha (A.) vitripennis, type 4; **Location:** continent: Europe; country: Montenegro; locality: Prokletje Mts.; verbatimSRS: WGS84; decimalLatitude: 42.550042; decimalLongitude: 19.825639; **Identification:** identifiedBy: Keresztes, L.; **Event:** eventDate: 02-05-2022; eventRemarks: leg. Dénes, A.

#### Taxon discussion

Type 1 corresponds to the “normal” shape of the inner gonostylus, according to Geiger (1985) and it is the most widespread amongst male individuals in the western part of Europe. The apical part of outer gonostylus tapered, ending in a single point, lacking protrusion or humps or teeth on its upper surface (Fig. 4a and b). Amongst this type, it is also important to point to the most frequently detected narrow and straight inner branch of parameres, slightly curved in the distal end (Fig. 4c), but also a number of individuals, even in the same population, where the inner branch of parameres are divergent and highly sinuous in shape (Fig. 4d), for example, in several individuals collected from Lafnitz, Austria. The parameral lobes are rounded, protruded or almost straight at their distal end (Fig. 4a, c and d).

Our data show that type 1 of the inner gonostylus is widespread also in the central and eastern parts of Europe and it was detected in the hilly regions from Germany, Spain, Austria, Croatia, Romania and also in the Balkan Range Mountains (Stara Planina), Bulgaria. Out of the 201 male individuals, 157 specimens belong to this type, also representing the majority of male individuals investigated by us (roughly 78%).

Type 2, distinct from type 1, has a bifurcated outer gonostylus tip. The upper arm is shorter, always wider, more or less rounded or triangular, while the lower point is narrower, longer and almost thorn-like (Fig. 4d, e and h). Male individuals examined by us from Germany (surrounding areas of the City of Kassel), Romania (Banat Region) and Albania (Korce Region) belong to this type, representing 9% of the total individuals collected by us. Inner branch of parameres almost parallel, slightly sinuous at the distal end. The parameral lobes similar to type 1.

Type 3 represents individuals with outer gonostlylus ending in two points, similar to type 2, but the upper extension of the distal end is well developed, rounded and hump-like, while the lower part is more pointed and thorn-like (Fig. 4f). This type was completely absent from the material collected in the western part of Europe and we could only identify, in this case, a few specimens collected at high altitudes, in Bulgaria (Pirin Mountains, 1780 m) and Greece (Olympus Mountains, 2100 m) (less than 2% from the total individuals examined by us). Type 3 was collected from higher elevations, above 1500 m (Table 1). Inner branch of parameres and parameral lobes similar to type 1 and type 2.

Type 4 corresponds with the outer gonostylus sharply curved inwards and rounded at the distal end. A more or less well-developed tooth-like extension can often be distinguished in its lower corner (Fig. 4g). However, this type is difficult to separate from the previous type 3, because we encountered a series of transitional forms amongst individuals collected in the Balkan Range, which also resembles type 2 in some cases (Fig. 4h). Type 4 was identified amongst individuals collected in Albania, Montenegro and Georgia and it is the dominating type in the Balkan area, representing 9% of the total individuals examined by us. Inner branch of parameres are more slender and sinuous. Parameral lobes with well-developed distal lobes.

## Identification Keys

### Key to West Palearctic males of Antocha (Antocha)

**Table:** 

1	Wing without a pronounced anal lobe	Antocha (Orimargula)
–	Wing with a pronounced anal lobe	2
2	Inner branch of parameres wide, with 2 teeth-like points at distal end	A. (A.) libanotica
–	Inner branch of parameres slender and simple ending with one point at distal end	3
3	Outer gonostylus gradually narrowed at distal end, ending in one or two distinct points, slightly curved downwards, inner branch of parameres slender and sinuous, sometimes parallel with the aedeagal axis	A. (A.) vitripennis
–	Outer gonostylus sharply curved down at distal end, rounded, inner branch of parameres straight and parallel with the aedeagal axis	4
4	Parameral lobes weakly developed	A. (A.) hirtipes
–	Parameral lobes well developed	5
5	Parameral lobes wide at base, divergent distally	A. (A.) phoenicia
–	Parameral lobes narrow at base, parallel distally with aeadeagal axis	A. (A.) staryi sp. nov.

## Discussion

Antocha (A.) vitripennis has a long-debated taxonomy. The species was first described from the northern part of Germany (most probably around Stolberg) by Meigen (1830) as *Limnobiavitripennis* (syntypes deposited in the Muséum national d’Histoire naturelle, Paris (France), Diptera Collection (ED), specimen identification code MNHN-ED-ED2450), based on translucent or opalescent wings (vitri - window, pennis - wing, lat.), later transferred to the subgenus Antocha of the genus *Antocha* by Osten Sacken (1860).

The species is widely distributed in the whole West Palearctic area (Oosterbroek 2023), but also detected from Bashkortostan Republik in Russia (East Palearctic) (Kolcsár et al. 2021).

Antocha (A.) fulvescens Lackschewitz, 1940, very close to A. (A.) vitripennis, was described by Lackschewitz (1940) from Albania in 1940 and later identified also from France (Geiger 1985), based on a generally lighter colour of the specimens, shape of the outer gonostylus ending in two points and the divergent position of the inner branch of the parameres relative to the body axis. Based on comparative analyses of a large amount of material of *Antocha* from Europe, Geiger revised the taxonomic status of A. (A.) fulvescens and synonymised it with A. (A.) vitripennis because of the high variability of the male genital structures of individuals in sympatric populations, not only in the Balkan Range, but also from different European countries, like Switzerland, France and Germany (Geiger 1985). However, Mendl still argued on the possibility of the presence of more subspecies of A. (A.) vitripennis in Europe, especially in the Balkan Range (Mendl 1979).

Our result supports the work of Geiger (1985) on the presence of the single, but highly variable A. (A.) vitripennis in Europe, based on a large amount of fresh material collected from different parts of Europe. Examination of 201 male genital structures reveals highly-different morphological types of the outer gonostylus (here noted as type 1 to type 4), which is not followed by any other detectable differences in the other parts of the male terminalia. The geographic distribution of the identified differences does not draw out any clear clinal pattern (Fig. 1). The widely-distributed type 1 is very frequent in Western Europe and Central Europe and represented the majority of individuals investigated by us (78%), but were present sporadically also in the Balkan Range, mostly along some intermittent waters in the hilly area around the Plitvice Lakes (Croatia) and the Eastern Stara Planina Mountains (Bulgaria). Type 2, represents a morphological variability of male genital structures similar to A. (A.) fulvipennis (sensu Lackschewitz 1940) and was identified by us not only in the Balkan area (Albania), but also in Germany and Romania. Type 3, however, is a particular case of highly different morphology of the outer gonostylus, distinct from type 1 or type 2, collected only from the mountainous areas of Bulgaria and Greece, up to 1500 m altitude, which is also unusual for A. (A.) vitripennis, a species frequently collected at lower hilly or mountainous regions, but missing from high altitudes. Unfortunately, the small number of individuals (2 males) collected during our investigation did not allow us to support the presence of a separate A. (A.) vitripennis taxa in the Balkans, but points to the necessity for a more comprehensive revision of the subgenus in its whole range. Type 4 is even more difficult to appreciate, but it is typical for a large number of individuals collected in the Southern Balkans, but also in the case of one male from Georgia and supports a wide range of transitional forms between type 2 and type 3, also highly similar with A. (A.) hirtipes Savchenko, 1971, described from Georgia by Savchenko in 1971. The detection variability of type 4 and its similarity to A. (A.) hirtipes makes the status of the later taxa in Georgia questionable. The single male individual of A. (A.) vitripennis collected by us in Georgia is not clearly different from the individuals of A. (A.) vitripennis from the Balkan area. However, in the original description of A. (A.) hirtipes (Savchenko 1971), the parameral lobes are not figured, but are clearly well developed in the male individuals collected by us. Based on these taxonomic challenges and also the recent discovery of A. (A.) vitripennis in the East Palearctic area (Kolcsár et al. 2021), a taxonomic revision of all members of the subgenus is highly recommended.

In conclusion, the subgenus Antocha is represented by five species in the West Palearctic area. Except for the high mountainous Antocha (A.) libanotica, which has a highly different shape of inner branch of parameres, ending in two points, the rest of the West Palearctic species, A. (A.) hirtipes, A. (A.) phoenicia and A. (A.) vitripennis, including the recently discovered A. (A.) staryi sp. nov., are mostly hilly species, highly similar in general habitus and shape of the male and female terminalia. The taxonomic status of type 3 of A. (A.) vitripennis remains challenged and this particular morphology of the outer gonostylus was detected in some few individuals only, collected at higher elevations in the Pirin Mountains, Bulgaria and Olympus Mountains, Greece. Further, morphological investigation supported by molecular data is highly recommended to test the taxonomic status of this particular morphology, as well as for the other members of the subgenus Antocha.

Despite the extremely high morphological variability of the male outer gonostylus in the case of A. (A.) vitripennis, a series of small, but constant details on other male genital structures, including the parameres and aedeagus, allow us to separate the five species of the subgenus A. (Antocha), supported also by information from literature (Savchenko 1971, Thomas and Dia 1982).

## Supplementary Material

XML Treatment for Antocha (Antocha) staryi

XML Treatment for Antocha (Antochа) vitripennis

## Figures and Tables

**Table 1.** Sampling sites of Antocha (Antocha) vitripennis from the West Palaearctic Region, included in this study, with number on individual males, localities, coordinates, altitude and name of the collectors. Morpho-types abbreviation sorted by country: ***AV-TYPE 1*** – A. (A.) vitripennis, type 1; ***AV-TYPE 2*** – A. (A.) vitripennis, type 2; ***AV-TYPE 3*** – A. (A.) vitripennis, type 3; ***AV-TYPE 4*** – A. (A.) vitripennis, type 4.

Species/Type	Male	Country	Region	Longitude	Latitude	Data	Collectors
***Av* - type 1**	25	Austria	Burgenland	47.373709°N	16.010401°E	19.06.2012	Graf, W.
***Av* - type 1**	5	Austria	Wienna hills	48.136847°N	16.098223°E	15.06.2012	Graf, W.
***Av* - type 1**	4	Bulgaria	Stara Planina	42.784125°N	25.912326°E	01.06.2013	Kolcsár, L- P, Török, E.
***Av* - type 1**	7	Croatia	NP Plitvicka Jezera	44.925833°N	15.619167°E	30.06.2008	Ivkovic, M.
***Av* - type 1**	6	Croatia	Omiši Forests	43.437297°N	16.757657°E	19.07.2005	Ivkovic, M.
***Av* - type 1**	2	Germany	Danube lowland	49.026846°N	12.075860°E	13.07.2013	Graf, W.
***Av* - type 1**	22	Romania	Rodnei Mts.	47.423995°N	24.548807°E	26.07.2003	Keresztes, L.
***Av* - type 1**	33	Romania	Harghita Mts.	46.412488°N	25.745437°E	15.07.2001	Keresztes, L.
***Av* - type 1**	37	Romania	Ciucaș Mts.	45.534297°N	25.836821°E	21.07.2004	Keresztes, L.
***Av* - type 1**	7	Romania	Trascaului Mts.	46.565262°N	23.675828°E	06.06.2001	Keresztes, L.
***Av* - type 1**	8	Romania	Clujului Hills	46.755529°N	23.509461°E	24.08.2004	Keresztes, L.
***Av* - type 1**	1	Spain	Serrania de Cuenca	39.503257°N	(-)1.900691°E	04.08.2009	Martinez, J.
***Av* - type 2**	1	Albania	Korce	41.016258°N	20.513618°E	03.05.2019	Keresztes, L.
***Av* - type 2**	12	Germany	Eifel hills	49.755204°N	6.735459°E	05.08.2007	Neu, P.
***Av* - type 2**	6	Romania	Cerna Mts.	45.256819°N	22.814289°E	10.08.2004	Bálint, M.
***Av* - type 3**	2	Bulgaria	Pirin Mts.	41.768952°N	23.426897°E	19.08.2003	Pauls, S.
***Av* - type 3**	1	Greece	Olympus Mts.	40.079097°N	22.373471°E	14.07.2012	Rákosy, L.
***Av* - type 4**	13	Albania	Domosdova Plain	41.075075°N	20.498387°E	01.05.2019	Keresztes, L.
***Av* - type 4**	1	Georgia	North Caucaus	43.122052°N	42.750700°E	18.07.2012	Graf, W.
***Av* - type 4**	5	Montenegro	Prokletje Mts.	42.550042°N	19.825639°E	02.05.2022	Dénes, A.
